# A structural basis for the strain-dependent UDP-sugar specificity of glycosyltransferase C from the *Limosilactobacillus reuteri* accessory secretion system

**DOI:** 10.1107/S2059798325008782

**Published:** 2025-11-05

**Authors:** Ryan Griffiths, Hans Pfalzgraf, Dimitris Latousakis, Gareth Ashworth, Changjiang Dong, Andrew Hemmings, Nathalie Juge

**Affiliations:** a Food, Microbes and Health Quadram Institute Bioscience Norwich United Kingdom; b School of Biological Sciences University of East Anglia Norwich United Kingdom; c School of Medicine University of East Anglia Norwich United Kingdom; d School of Chemistry, Pharmacy and Pharmacology University of East Anglia Norwich United Kingdom; e International Research Center for Food and Health, College of Food Science and Technology Shanghai Ocean University Shanghai China; Station Biologique de Roscoff, France

**Keywords:** accessory secretion systems, glycosyltransferases, crystal structure, GtfC

## Abstract

The accessory secretion (aSec) system is a protein export pathway that is uniquely present in Gram-positive bacteria and is dedicated to the secretion of large, glycosylated cell wall-anchored adhesins called serine-rich repeat proteins (SRRPs). Strain-specific glycosylation of SRRPs has previously been reported in *Limosilactobacillus reuteri* and attributed to GtfC, a glycosyltransferase belonging to family 113, with *Lr*GtfC_100-23_ from *L. reuteri* rat strain 100-23C showing specificity for UDP-Glc, while *Lr*GtfC_53608_ from *L. reuteri* pig strain ATCC 53608, which differs at only ten amino-acid positions, shows a preference for UDP-GlcNAc. However, the structural basis underpinning GtfC sugar-donor specificity remains unclear. Here, we report X-ray crystal structures of the tetrameric *Lr*GtfC_100-23_ in the apo form and its complexes with UDP and with the noncognate sugar donor UDP-*N*-acetylglucosamine (UDP-GlcNAc). Analysis of the *Lr*GtfC_100-23_ structures identified candidate residues implicated in donor-sugar substrate specificity, which were supported by site-directed mutagenesis. Reciprocal swaps of candidate residues combined with thermal shift assays revealed that the W240C variant of *Lr*GtfC_100–23_ could bind both UDP-sugar donors, while the P243S variant of *Lr*GtfC_53608_ became specific for UDP-Glc, opening the door for glycoengineering approaches in bacteria.

## Abstract 2

X-ray crystal structures of glycosyltransferase C from the *Limosilactobacillus reuteri* 100-23 (*Lr*GtfC_100-23_) accessory secretion system were determined in its apo form and in complexes with UDP and with UDP-*N*-acetylglucosamine, which revealed candidate residues involved in strain-specific recognition of UDP-sugars. Site-directed mutagenesis of these residues led to a switch of the UDP-sugar binding specificities of *Lr*GtfC_100-23_ and the highly homologous *Lr*GtfC from *L. reuteri* ATCC 53608.

## 1. Introduction

*Limosilactobacillus reuteri* (previously *Lactobacillus reuteri*) is a Gram-positive bacterium present in the gut of a diverse range of vertebrates (Walter *et al.*, 2011 ▸; Sinkiewicz & Ljunggren, 2008 ▸; Mu *et al.*, 2018 ▸; Zheng *et al.*, 2020 ▸). *L. reuteri* displays a co-evolutionary relationship with its hosts and has beneficial effects in humans and animals (Cleusix *et al.*, 2007 ▸; Mu *et al.*, 2018 ▸). The mechanisms of adaptation of *L. reuteri* to the host have been extensively studied (Duar *et al.*, 2017 ▸; Frese *et al.*, 2011 ▸, 2013 ▸; Lin *et al.*, 2018 ▸; Walter *et al.*, 2011 ▸). In mice, the capacity of *L. reuteri* to form forestomach epithelial biofilm has been attributed to serine-rich repeat proteins (SRRPs; Frese *et al.*, 2013 ▸).

SRRPs are glycoproteins that are transported to the cell surface by a dedicated accessory secretion (aSec) system present in Gram-positive bacteria (Szymanski, 2022 ▸; Nothaft & Szymanski, 2010 ▸; Li *et al.*, 2017 ▸; Latousakis & Juge, 2018 ▸). These glycoproteins have been studied extensively in pathogens such as *Staphylococcus aureus*, *Streptococcus gordonii* and *Streptococcus pneumoniae*, where they have been implicated in adhesion, aggregation, biofilm formation, immune modulation and virulence (Sanchez *et al.*, 2010 ▸; Chan *et al.*, 2020 ▸; Sequeira *et al.*, 2018 ▸; Zhou & Wu, 2009 ▸). The aSec glycosylation system encodes chaperones, acetyltransferases, a motor ATPase, membrane translocon apparatus and a diverse number of glycosyltransferases (GTs) (Latousakis *et al.*, 2020 ▸; Rigel & Braunstein, 2008 ▸; Braunstein *et al.*, 2019 ▸). The secretion cargo undergoes *O*-glycosylation, where Ser/Thr residues are initially modified with GlcNAc moieties by a primary GtfA–GtfB complex (Bensing & Sullam, 2002 ▸; Shi *et al.*, 2014 ▸). GtfA is a GT4-type glycosyltransferase, while GtfB is a catalytically inactive chaperone with a tetrameric quaternary structure (Chen *et al.*, 2016 ▸; Cantarel *et al.*, 2009 ▸). Subsequent glycosylation is carried out by additional GTs, including GtfC, belonging to the GT113 family (https://www.cazy.org), also annotated Gtf3 in several streptococcal species (*S. pneumoniae*, *S. parasanguinis* and *S. mutans*), leading to diverse SRRP glycosylation profiles. The aSec GTs and associated glycosylation of the SRRP carrier have been shown to be critical for virulence, whereby deletion mutations of Gtf1 (a GtfA homolog) and Gtf3 greatly attenuated biofilm formation in streptococcal species (Rainey *et al.*, 2019 ▸; Zhu *et al.*, 2016 ▸). Previous work in *S. parasanguinis* also mapped several of the residues required for Gtf3 function. These include Arg179 and Lys246, which are required for nucleotide sugar-donor binding, and Leu320, which is required for oligomerization. Asp101 is involved in catalysis in accordance with a retaining GT mechanism (Zhu *et al.*, 2011 ▸). The glycosylation pathway of SRRPs has also been characterized in nonpathogenic strains such as *L. reuteri* strains 100-23C (from rodent) and ATCC 53608 (from pig) (Latousakis *et al.*, 2019 ▸; Sequeira *et al.*, 2018 ▸). After initial *O*-GlcNAc deposition by GtfAB, subsequent glycosylation by GtfC and other GTs leads to strain-specific glycosylation of *Lr*SRRP_100-23_ and *Lr*SRRP_53608_ with Hex-Glc-GlcNAc and di-GlcNAc moieties, respectively (Latousakis *et al.*, 2019 ▸, 2020 ▸; Sequeira *et al.*, 2018 ▸).

To gain insight into the strain-specific glycosylation of *Lr*SRRPs, we used a structural biology approach to investigate the UDP-sugar donor specificity of *L. reuteri* GtfC from *L. reuteri* strains 100-23C and ATCC 53608. We present the crystal structure of *Lr*GtfC_100-23_ in the apo form and in complexes with UDP and the noncognate sugar donor UDP-GlcNAc. Supported by mutagenesis, these structures reveal the key sugar-nucleotide binding residues underpinning strain-specific glycosylation of *L. reuteri* SRRPs.

## 2. Materials and methods

### 2.1. Protein expression and purification

The heterologous overexpression and purification of *Lr*GtfC_100-23_ (UniProt entry B3XPQ7, GTF3_LIMR1) was performed as described previously to produce a recombinant protein with an N-terminal 6×His affinity tag (His_6_-tag) and a thrombin cleavage site (Latousakis *et al.*, 2019 ▸). A similar methodology was used for the production of recombinant *Lr*GtfC_53608_ (UniProt entry F8KEJ1, GTF3_LIMR5), except that the protein was expressed using a pOPINF vector (Berrow *et al.*, 2007 ▸) with an N-terminal His_6_-tag and an HRV-3C protease cleavage site. *Lr*GtfC_53608_ was then purified by immobilized metal-affinity chromatography (IMAC) and His_6_-tag cleavage was performed at 4°C overnight using 1 unit of HRV-3C protease (Abdelkader & Otting, 2021 ▸). The cleavage product was then purified by size-exclusion chromatography (SEC) using a running buffer consisting of 200 m*M* NaCl, 20 m*M* Tris pH 7.9 and a HiLoad 16/600 Superdex 75 pg column (GE Healthcare) at a flow rate of 0.75 ml min^−1^. *Lr*GtfC_100-23_ and *Lr*GtfC_53608_ recombinant proteins were concentrated for crystallography or biochemical analysis by centrifugation using a Vivaspin 10 000 molecular-weight cutoff spin-filter at 4°C. The extinction coefficients used were 69 330 and 62 465 *M*^−1^ cm^−1^ for *Lr*GtfC_100-23_ and *Lr*GtfC_53608_, respectively.

### 2.2. Crystallization

Crystallization trials were performed at 289 K by sitting-drop vapor diffusion in 96-well 2-drop MRC plates using commercial screens (Molecular Dimensions) and an OryxNano robot (Douglas Instruments). A drop size of 0.5 µl containing the protein and screen solution in either a 1:1 or 1:2 ratio was equilibrated against 50 µl screen solution per reservoir. To produce crystals of the apoenzyme, crystallization screening was conducted with the purified His_6_-tagged *Lr*GtfC_100–23_ at a concentration of 10 mg ml^−1^ in 20 m*M* Tris pH 8.0, 150 m*M* NaCl. Promising crystals formed in the PACT *premier* screen (Newman *et al.*, 2005 ▸), and the pH and PEG concentration of one of the conditions was optimized to 0.2 *M* potassium thiocyanate, 0.1 *M* bis-Tris propane pH 7.3, 18%(*w*/*v*) PEG 3350. After ten months, intergrown plates were sheared from a crystal before harvesting. For crystallization of the UDP-bound enzyme, the same His_6_-tagged apo *Lr*GtfC_100-23_ sample was incubated with 1 m*M* UDP (final protein concentration of 9 mg ml^−1^) before setting up crystallization in a 1:1 ratio of protein + UDP to screen solution. Crystals appeared after 14 days using a solution consisting of 0.1 *M* bis-Tris pH 7.5, 0.2 *M* potassium thiocyanate, 20%(*w*/*v*) PEG 3350. Crystals of apo and UDP-bound enzyme were harvested using mounted LithoLoops (Molecular Dimensions) and transferred into drops containing well solution with glycerol added to 30%(*v*/*v*) for cryoprotection before flash-cooling in liquid nitrogen. Finally, for the UDP-GlcNAc-bound enzyme, purified untagged *Lr*GtfC_100-23_ in 20 m*M* HEPES pH 7.1, 100 m*M* NaCl buffer was mixed with a solution of UDP-GlcNAc disodium salt (Biosynth) in the same buffer to obtain a protein concentration of 11 mg ml^−1^ and a 100:1 ligand:enzyme molar ratio for crystallization screening, including the use of JCSG-*plus* (Newman *et al.*, 2005 ▸). A crystal from a precipitant solution consisting of 0.2 *M* ammonium citrate dibasic, 20%(*w*/*v*) PEG 3350 was harvested seven days later; reservoir solution with 20% glycerol for cryoprotection was added before flash-cooling in liquid nitrogen.

### 2.3. X-ray data collection and crystal structure analysis

X-ray diffraction data collection was performed at Diamond Light Source. For the apo structure, 3600 frames of 0.1° rotation were collected on beamline I04 using an EIGER2 XE 16M (Dectris) detector with an exposure time of 0.02 s and a wavelength of 0.9795 Å. For the UDP-bound structure, 2400 frames of 0.15° rotation were collected on beamline I24 using a PILATUS3 6M (Dectris) detector. For the UDP-GlcNAc-bound structure, a CdTe EIGER2 9M (Dectris) detector was employed and only the first 1200 frames were used to avoid radiation damage. Data reduction for the apo and UDP-GlcNAc-bound structures was performed with *DIALS*, while that for the UDP-bound structure was performed with *xia*2 (Winter *et al.*, 2013 ▸).

Phases were determined for the structure of apo *Lr*GtfC100-23 by molecular replacement using *Phaser* (McCoy, 2007 ▸) from the *Phenix* software suite (Liebschner *et al.*, 2019 ▸) with the structure of a single subunit of the homologous glycosyltransferase 3 from *S. parasanguinis* (PDB entry 3qkw; Zhu *et al.*, 2011 ▸) as a search model, as suggested by *Phyre*2 (Kelley *et al.*, 2015 ▸). Phases for the *Lr*GtfC_100-23_ complexes with UDP and UDP-GlcNAc were then determined by molecular replacement using a single subunit of the apo *Lr*GtfC_100–23_ structure to obtain a solution for the molecular tetramer.

All structures were refined using a combination of *phenix.refine* (Afonine *et al.*, 2012 ▸) combined with manual rebuilding in *Coot* (Emsley & Cowtan, 2004 ▸). Structures were then subject to several rounds of refinement with *REFMAC*5 (Murshudov *et al.*, 2011 ▸; Yamashita *et al.*, 2023 ▸), again combined with manual model refinement using *Coot*. Data-collection and structure-refinement statistics are provided in Table 1 ▸.

### 2.4. Site-directed mutagenesis

Site-directed mutagenesis was carried out by PCR using a method adapted from Edelheit *et al.* (2009 ▸). Briefly, forward and reverse primers (Supplementary Table S1) were used in separate single-primer reactions in parallel using Platinum SuperFi II high-fidelity polymerase (ThermoFisher) and an annealing temperature of 60°C. The resulting PCR products containing parental plasmid DNA and either the 5′–3′ or the 3′–5′ amplicons were then mixed in a 1:1 ratio. The mixture was denatured at 95°C for 5 min and cooled using a sequential thermal ramp from 90°C to 25°C (90°C for 2 min, 80°C for 2 min, 70°C for 2 min, 60°C for 2 min, 50°C for 2 min, 40°C for 2 min, 30°C for 2 min and 25°C for 2 min), promoting random annealing of plasmid strands. The PCR products were digested with DpnI, leaving only the annealed mutated plasmids. The digestion product was used to transform *Escherichia coli* BL21(DE3) cells by heat-shock transformation. Unless stated otherwise, reagents and restriction enzymes were obtained from New England Biolabs.

### 2.5. Thermal shift assays

Thermal shift assays were conducted using a StepOnePlus Real Time PCR system (ThermoFisher). Assays were performed in a final volume of 25 µl using 20 µ*M* detagged proteins and SYPRO Orange dye (Merck) at a 10× final concentration, as described previously (Latousakis *et al.*, 2019 ▸). Assays were conducted in 50 m*M* Tris pH 7.5, 100 m*M* NaCl in the absence or presence of 3 m*M* UDP-Glc (Carbosynth) or 3 m*M* UDP-GlcNAc (Carbosynth). A thermal ramp from 25 to 90°C was used with 1°C stepwise increases. Samples (*n* = 4, technical duplicates from two different protein preparations) were randomized in a MicroAmp 96-well plate (ThermoFisher) and the resulting first derivative of the fluorescence intensity curve (plotted against temperature) was used to calculate the melting temperature (*T*_m_) by identifying the peak of the derivative curve in *Microsoft Excel*. Tests for significance of the observed change in *T*_m_ for a variant in the presence of a ligand relative to that observed for the variant in the absence of the ligand were carried out using Student’s *t*-test. Results are presented as the means ± standard deviations (SD). Significance was defined at *p*-values of <0.05.

### 2.6. Predicted structure for *Lr*GtfC_53608_

Predicted structures of *Lr*GtfC_100–23_ (corresponding to UniProt entry B3XPQ7) and *Lr*GtfC_53608_ (corresponding to UniProt entry F8KEJ1) were taken from the AlphaFold Protein Structure Database (Jumper *et al.*, 2021 ▸; Varadi *et al.*, 2023 ▸).

## 3. Results and discussion

### 3.1. *Lr*GtfC_100-23_ has a GT-B fold and crystallized as a tetramer

**Figure 1:**
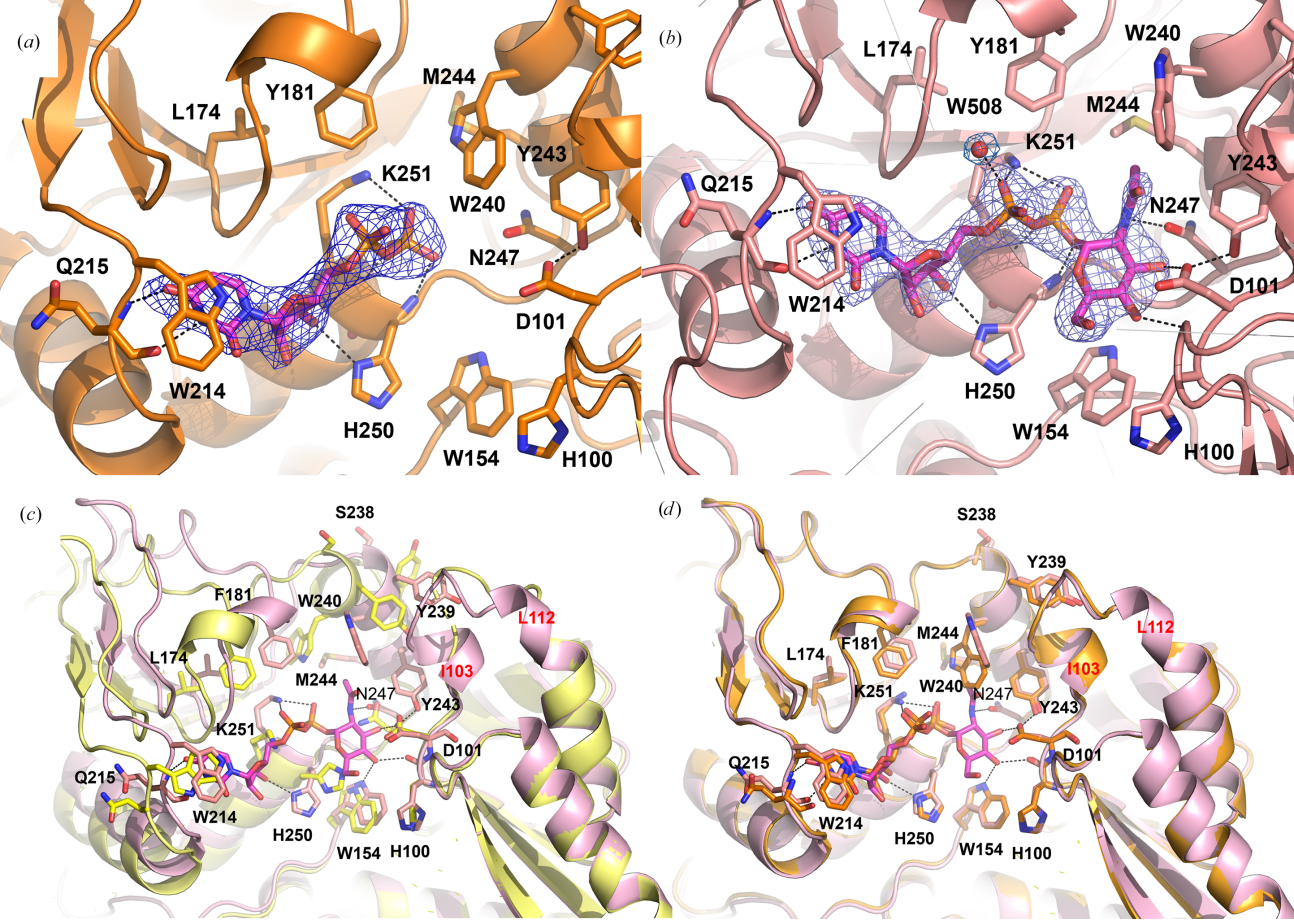
Crystal structures and conformational changes accompanying ligand-bound *Lr*GtfC_100-23_. (*a*, *b*) Details of the binding of UDP and the UDP-GlcNAc sugar donor (magenta C atoms), respectively, to *Lr*GtfC_100-23_. The ligands and neighboring residues (labeled) are shown in stick format with a cartoon representation of the enzyme. Polar interactions are indicated by black dashed lines. A ligand omit map calculated contoured at 3σ is shown as a blue mesh in both panels. Note that here and in subsequent panels, residues 1–67 have been removed for clarity and so the hydrogen-bond interaction of the α-phosphate of ligands with the side chain of Thr16 is not seen. (*c*, *d*) Conformational changes observed during ligand binding. (*c*) A superposition of the N-terminal acceptor-binding domains of the apo (yellow cartoon, yellow C atoms) and sugar donor-bound (magenta cartoon, magenta C atoms) structures. Bound UDP-GlcNAc is shown in stick format with a molecular-surface rendering. Residues 103 and 112 (red text) delimit a flexible region of polypeptide adjacent to the binding site in the apo structure. (*d*) A superposition of the sugar donor-bound (orange cartoon, orange C atoms) and UDP-bound (gray cartoon, gray C atoms) enzyme structures. Bound UDP is shown in stick format. For each structure selected residues are shown in stick format and with a selection of hydrogen-bond interactions indicated by black dashed lines.

Crystals of recombinant apo *Lr*GtfC_100-23_ (monomer molecular weight ∼38 kDa) in space group *P*2_1_2_1_2_1_ diffracted to 2.0 Å resolution and yielded a structure of the fully apo enzyme with an *R* factor of 21.3% (*R*_free_ = 25.6%). The average *B* factor for this structure was higher than observed for the structures of its complexes with sugar donor or UDP, with the more solvent-exposed C-terminal sugar-donor binding domains having greater mobility (Supplementary Fig. S1). Crystals of *Lr*GtfC_100–23_ grown in the presence of UDP in space group *P*2_1_ diffracted to 2.6 Å resolution and yielded a structure with an *R* factor of 22.6% (*R*_free_ = 27.9%). In this structure, three subunits were found to contain bound UDP (chains *A*, *B* and *C*), while subunit *D* was vacant (Fig. 1 ▸*a*). Attempts to soak crystals of the apoprotein using the preferred cognate sugar donor UDP-Glc led to crystal dissolution. X-ray data collected from crystals of *Lr*GtfC_100–23_ where UDP-Glc was added for co-crystallization resulted in structures with only UDP bound (data not shown). That no electron density could be identified for the sugar moiety is likely to be due to the enzymatic hydrolysis of UDP-Glc with water as the sugar acceptor that occurs over the long timescale of the crystallization experiments in the absence of the SRRP acceptor substrate (Brockhausen, 2014 ▸; Sindhuwinata *et al.*, 2010 ▸). While *Lr*GtfC_100-23_ binds the noncognate UDP-sugar UDP-GlcNAc more weakly than it does UDP-Glc (Latousakis *et al.*, 2019 ▸), co-crystallization with a 100-fold molar excess of UDP-GlcNAc gave crystals in space group *P*2_1_2_1_2_1_ which diffracted to 2.4 Å resolution. The resulting structure with an *R* factor of 19.0% (*R*_free_ = 22.3%) possessed two subunits in complex with UDP-GlcNAc (chains *B* and *C*), one subunit with UDP (chain *A*) and one vacant subunit (chain *D*) (Fig. 1 ▸*b*). In the case of chain *A*, the UDP molecule may have been present as an impurity or formed either through enzymatic hydrolysis, as seen for other GTs (Esposito *et al.*, 2018 ▸), or as a spontaneous degradation product of UDP-GlcNAc (Namboori & Graham, 2008 ▸). Despite its high sequence identity to *Lr*GtfC_100-23_, we were unable to crystallize recombinant *Lr*GtfC_53608_, whether in the apo form or in complex with either UDP or UDP-Glc.

Whilst *Lr*GtfC_100-23_ was found to crystallize non-isomorphously, all three crystal forms contained a single copy of the enzyme tetramer in the crystallographic asymmetric unit (Supplementary Figs. S2 and S3). The tetramer consists of two pairs of back-to-back dimers with outward-facing active sites. Three major folds have been identified for GTs: GT-A (two abutting β/α/β Rossmann-like domains, metal ion-dependent), GT-B (two facing β/α/β Rossmann-like domains with a central cleft) and GT-C (mainly membrane proteins with lipidic sugar donors) (Gloster, 2014 ▸). Here, *Lr*GtfC_100-23_ monomers displayed a classic GT-B fold consisting of similar N-terminal acceptor-binding and C-terminal donor-binding Rossmann-like domains, each with a central six-stranded β-sheet (Latousakis, MacKenzie *et al.* 2020 ▸). Interestingly, a tetrameric oligomerization (comprised of two GtfAB dimers) has been predicted for the GtfAB complex of aSec systems from *Streptococcus gordonii* and *S. pneumoniae* (Chen *et al.*, 2016 ▸, 2018 ▸). In these cases, the inactive GtfB subunit acts as the acceptor-binding component, whereas GtfA acts as the putative transferase. One model predicts that while one GtfAB unit binds and transfers the GlcNAc moiety to GspB (the *S. gordonii* SRRP), the other GtfAB unit can acquire new donor sugars and these units can, in turn, perform glycosylation of the SRR acceptors (Chen *et al.*, 2016 ▸). Based on our observations, it is tempting to speculate that *Lr*GtfC_100-23_ may adopt donor-vacant, donor-charged and acceptor-bound states which will rotate to glycosylate *Lr*SRRP_100-23_.

### 3.2. Conformational change accompanies binding of ligands to the apoenzyme

The active site, containing the conserved catalytic residue Asp101, is present as a cleft formed between the two domains of the enzyme. Binding of UDP-GlcNAc leads to a variety of structural changes ranging from local side-chain adjustments to a limited domain-closure motion and structural reorganization in flexible regions adjacent to the sugar-nucleotide binding site (Fig. 1 ▸*c*). The local structural rearrangements lead to an enhanced grasp of the ligand by active-site residues including Trp214 which packs against the uracil moiety, His250 which rotates to hydrogen-bond to the O3′ hydroxyl group of the ribose, Thr16 which hydrogen-bonds to the ribose O5 atom, and Lys251 which interacts with the α- and β-phosphate groups. These interactions are present in the complexes formed with both UDP and UDP-GlcNAc and are supplemented by two further hydrogen bonds between the uridine group and the main chain of Gln215.

The effect of ligand binding on domain packing was investigated using *DynDom* (Lee *et al.*, 2003 ▸) by comparing ligand-bound with apoenzyme structures. UDP-GlcNAc was found bound to the *B* and *C* monomers of the tetramer, while UDP was bound to chain *A*. The binding site of the chain *D* monomer was empty. Analysis of the chain *B* and *C* monomers revealed a small rotation (3.5°) between the donor-binding and acceptor-binding domains with a molecular hinge involving residues Met153 and Leu314 in the UDP-GlcNAc-bound structure (Fig. 1 ▸*c*). No significant translational component was observed. A similar rotational motion relates the UDP-bound and apoenzyme structures. The two ligand-bound conformations of *Lr*GtfC_100-23_ are more similar (r.m.s.d. of 0.29 Å) and no rigid-body motion relating them was detected (Fig. 1 ▸*d*). Loss of UDP followed by domain motion presumably serves to open the active site for product diffusion. GT-B folds often exhibit a global domain movement upon substrate binding, with open-to-closed conformational transitions typically in the range 10–12° (Bolam *et al.*, 2007 ▸; Chang *et al.*, 2011 ▸). The ∼4° rotation observed for *Lr*GtfC_100-23_ is relatively low in comparison. This motion is not sugar-specific in that it is observed in both the UDP- and UDP-GlcNAc-bound structures and has been reported previously for other enzymes (Hu *et al.*, 2003 ▸; Chang *et al.*, 2011 ▸).

The primary outcome of the binding of UDP-GlcNAc to the apoenzyme is to lead to a remodeling of the polypeptide in the region of the sugar moiety. Rotation of the acceptor-binding domain following ligand binding brings residues Ile103–Leu112 into contact with residues Ser238–Asn247 of the donor-binding domain, leading to a restructuring of these regions on sugar-nucleotide binding (Fig. 1 ▸*c*, Supplementary Fig. S4). While adopting a random-coil conformation characterized by high temperature factors in the N-domain of the apoenzyme structure, residues 103–112 transition to form an ordered helix–loop–helix motif. Changes also occur in the donor-binding domain, whereby residues Lys241–Leu246 undergo a transition from random coil to α-helix. As a result of this transformation, the indole group of Trp240 is rotated and moves nearly 7 Å to point towards the sugar ring of the ligand and, together with the side chains of Met244 and Phe181, contributes to a shallow hydrophobic pocket on the surface of the donor-binding domain. The rotation of the donor-binding domain also results in movement of the side chain of Phe181 towards Trp240, contributing to compression of this pocket. The *N*-acetyl methyl group of the sugar moiety pokes into this pocket on the surface of the donor-binding domain. The remodeling of C-domain residues 238–247 also results in movement of the outward-facing and solvent-exposed side chain of Tyr243 by more than 10 Å to move into a position in the active-site cleft where it can hydrogen-bond to the catalytic aspartate Asp101, in turn hydrogen-bonding to the sugar O3′ hydroxyl. Further direct hydrogen bonds are formed from the sugar O4′ hydroxyl to the carbonyl group of His100 and from the Asn247 side chain to the sugar *N*-acetyl N atom. It appears reasonable to assume that the binding of a cognate UDP-glucose sugar donor would lead to a specific hydrogen bond to the O2′ hydroxyl. Glycosyltransferases of both the GT-A and GT-B classes are frequently found to have flexible regions close to the sugar-nucleotide binding site that are postulated to play an important role during catalysis (Qasba *et al.*, 2005 ▸; Lairson *et al.*, 2008 ▸).

In contrast to the conformational changes occurring on ligand binding to the apoenzyme, loss of the sugar moiety leading to the UDP-bound state is accompanied by only minor structural adjustments (Fig. 1 ▸*d*, Supplementary Fig. S4). In this process the side chain of Trp240 relaxes on loss of the *N*-acetyl methyl group of the sugar donor and a shift in indole-group position is achieved by rotation about its side-chain χ_1_ and χ_2_ torsion angles. A corresponding shift, albeit small (0.5 Å), in the side chain of Phe181 away from the site of sugar binding is also observed.

### 3.3. *Lr*GtfC_100-23_ shares structural similarity with aSec glucosyltransferases from streptococci

The best matches for the structure of *Lr*GtfC_100–23_, as identified by *DALI* (Holm, 2022 ▸), both with *Z*-scores of 45.2, were the GT113 family enzymes Gtf3 from *S. parasanguinis* FW213 (PDB entry 3qkw; *Sp*Gtf3_FW213_; Zhu *et al.*, 2011 ▸) and GtfC from *S. agalactiae* (PDB entry 4w6q; *Sa*GtfC; Zhu *et al.*, 2015 ▸). Superposition of *Lr*GtfC_100-23_ with these proteins resulted in an r.m.s.d. of 0.77 Å over 275 C^α^ atoms and a sequence identity of 42% with *S. parasanguinis* Gtf3 and an r.m.s.d. of 0.86 Å over 268 C^α^ atoms and a sequence identity of 40% with *S. agalactiae* GtfC (Supplementary Fig. S5). Gtf3 from *S. parasanguinis* FW213 and GtfC from *S. agalactiae* COH1 are both glucosyltransferases (Zhu *et al.*, 2011 ▸, 2015 ▸). The next highest hit was to the structure of the *N*-acetyl­glucosamine transferase MshA from *Corynebacterium glutamicum* (PDB entry 3c4v; Vetting *et al.*, 2008 ▸), with a *Z*-score of 22.0. The corresponding r.m.s.d.. of 3.30 Å over 305 C^α^ atoms reflected a sequence identity of 13% and marked a significant decrease in relatedness among structural homologs.

Superposition of *Lr*GtfC_100-23_ with *Sp*Gtf3_FW213_ (PDB entry 3qkw) identified residues Lys180, Trp214 and Lys251 in *Lr*GtfC_100-23_, corresponding to Arg179, Tyr211 and Lys246 in *Sp*Gtf3_FW213_, within the UDP-binding site of Gtf3 (Supplementary Fig. S6). These basic residues are proposed to mediate the negative charge of the diphosphate, and their mutation produces inactive enzyme (Zhu *et al.*, 2011 ▸). Trp214 stacks above the uracil ring of UDP and the equivalent residue Tyr211 was found to be necessary for efficient substrate binding (Zhu *et al.*, 2011 ▸). A region within the α6 helix of the acceptor-substrate domain of *Sp*Gtf3_FW213_ that corresponds to residues Tyr319, Phe320, Lys322 and Lys323 in *Lr*GtfC_100-23_ was proposed to be involved in protein oligomerization, and their mutation led to reduced enzymatic activity (Zhu *et al.*, 2011 ▸). These residues also form part of the dimer interface in the *Lr*GtfC_100–23_ structure (Supplementary Fig. S7) and are likely to play a similar role. GtfC from *S. agalactiae* (*Sa*GtfC) contains a loop at positions 106–111 that was shown to be essential for enzymatic activity and acceptor-substrate binding, whereby deletion abolishes GT activity (Zhu *et al.*, 2015 ▸). The *Lr*GtfC_100–23_ MFESNR sequence corresponds to MFDGNF in *Sa*GtfC and these residues adopt very similar conformations in the two structures, highlighting conserved N-terminal residues contributing to active-site regions involved in sugar-moiety binding. Thus, the sequences and structures of *Lr*GtfC_100–23_, *Sp*Gtf3_FW213_ and *Sa*GtfC are similar, and the enzymes have related UDP-binding, oligomerization and donor substrate-binding characteristics.

### 3.4. Molecular and structural basis of *Lr*GtfC substrate specificities

**Figure 2:**
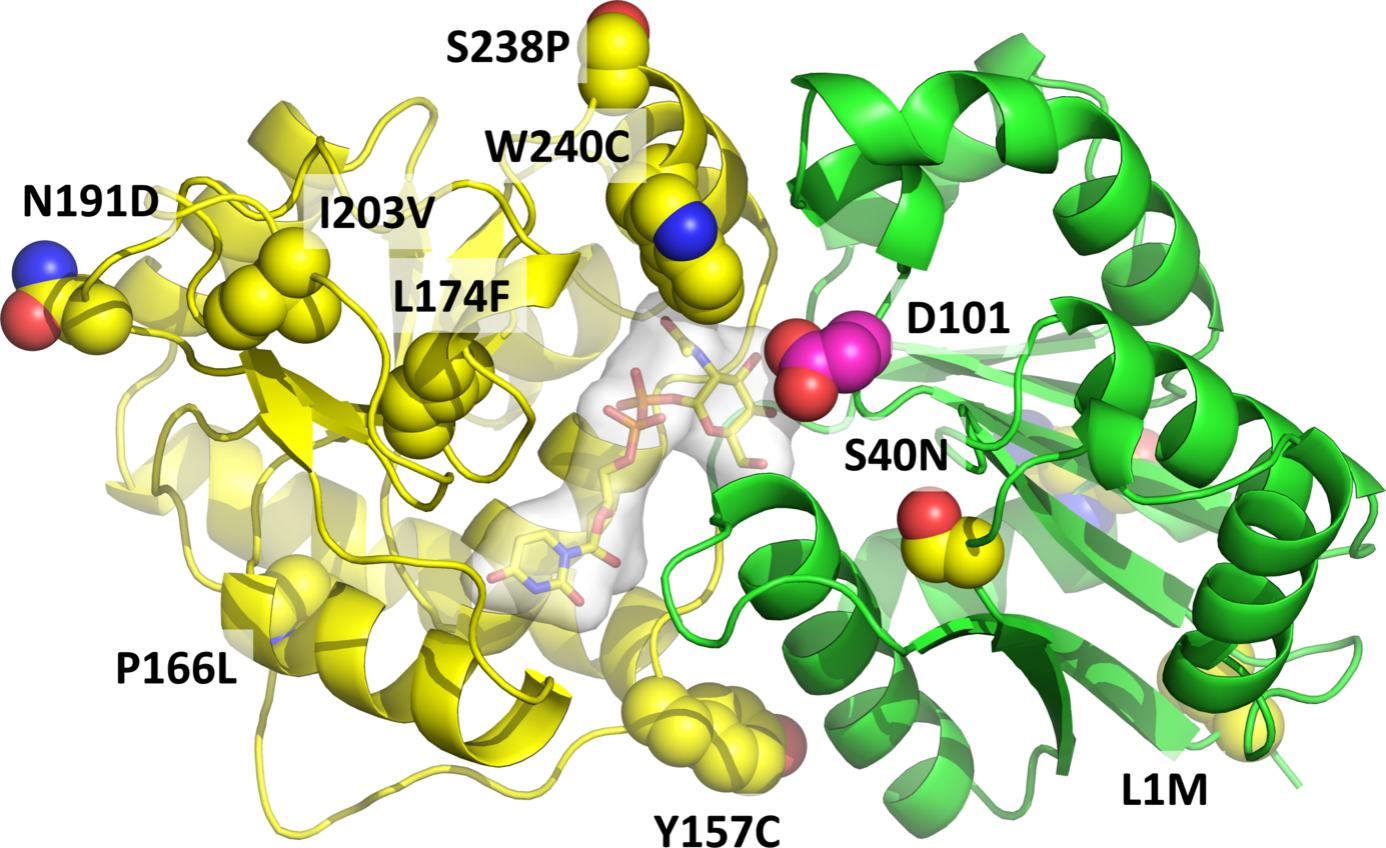
Locations of residue differences with *Lr*GtfC_53608_ mapped onto the crystal structure of sugar donor-bound *Lr*GtfC_100-23_. The enzyme is shown in a cartoon format with the N-terminal sugar acceptor-binding domain colored green and the C-terminal sugar donor-binding domain in yellow. Bound UDP-GlcNAc is shown in stick format with a molecular-surface rendering in white. Locations of residues of *Lr*GtfC_100-23_ which differ from those in *Lr*GtfC_53608_ are shown in sphere representation with C atoms colored yellow and the substitution labeled. Residue Asn331 in the N-terminal domain of *Lr*GtfC_100-23_, which is a lysine in *Lr*GtfC_53608_, is found at the back of the molecule in the orientation shown and is obscured in the image. The presumed catalytic aspartate, Asp101, is shown with C atoms colored magenta.

Recombinant *Lr*GtfC_53608_ could not be crystallized under any of the conditions tested despite having ∼97% sequence identity to *Lr*GtfC_100-23_ (Supplementary Fig. S8). The *AlphaFold*2-predicted structure of *Lr*GtfC_53608_ was superimposed on the crystal structure of *Lr*GtfC_100-23_ in complex with UDP-GlcNAc, showing small differences in residue side-chain positions in the UDP-sugar binding domain but no major conformational changes (Supplementary Fig. S9). The backbone of the *AlphaFold*2-predicted model of *Lr*GtfC_100-23_ only differed significantly in its low-confidence regions compared with the crystal structures. The conformations of the side chains in the predicted active site were most similar to those in the UDP-GlcNAc-bound crystal structure based on residues Trp240, Asn247, Asn249 and His250. At the sequence level, of the ten residues which differ between the two proteins, Leu1, Ser40 and Asn331 (in *Lr*GtfC_100-23_) are found within the N-terminal region dedicated to acceptor recognition and thus are considered unlikely to determine the donor specificity of the enzyme (Fig. 2 ▸). Of the remaining seven residues, Pro166, Asn191 and Ile203 are positioned on the outer face of the protein, distant from the donor substrate, and so were also deemed unlikely to be directly involved. Tyr157 is positioned on the interdomain polypeptide but is more than 15 Å away from the ribose ring of the substrate and so was similarly discounted. The remaining three residues were considered candidates for donor-substrate specificity determinants as they either lie in the proximity of the bound noncognate sugar donor (Trp240), are in regions of polypeptide that are remodeled on ligand binding (Ser238) or are conserved in functionally characterized glucosyltransferases (Leu174) (Fig. 2 ▸).

Based on this analysis, variants S238P, W240C and L174F of *Lr*GtfC_100-23_ and the corresponding variants, P243S, C245W and F179L, of *Lr*GtfC_53608_ were generated by site-directed mutagenesis and purified by affinity chromatography (Supplementary Fig. S10). In addition, variants of both wild-type enzymes in which the presumed catalytic aspartate residue (Asp101 in *Lr*GtfC_100-23_ and Asp106 in *Lr*GtfC_53608_) was replaced by an alanine were also generated to prevent possible hydrolysis of UDP-sugar substrates upon binding. The UDP-sugar specificities of the wild-type and variant proteins were tested using a thermal shift assay. Each variant was subjected to a thermal gradient from 25°C to 90°C in the absence or presence of UDP-Glc or UDP-GlcNAc (Supplementary Fig. S11). The quantification of mean thermal shifts (Δ*T*_m_), determined from the derivative data, are reported in Supplementary Table S2 and the corresponding probability of each shift relative to that observed for the same variant in the absence of ligand is presented in Table 2 ▸. The similarity in measured *T*_m_ values for wild-type and D101A (D106A in *Lr*GtfC_53608_) variant proteins suggests that spontaneous hydrolysis of the ligands did not occur over the time course of the thermal shift assay experiments.

Wild-type *Lr*GtfC_100-23_ showed a significant positive thermal shift (*p* < 0.0001) upon addition of its cognate sugar, UDP-Glc, while the addition of UDP-GlcNAc did not significantly affect the thermal stability (*p* = 8733) (Table 2 ▸, Supplementary Table S2). In contrast, *Lr*GtfC_100-23_ W240C showed a highly significant positive thermal shift in the presence of both UDP-GlcNAc or UDP-Glc (*p* < 0.0005). A significant but less pronounced effect was observed for the *Lr*GtfC_100-23_ S238P variant (*p* < 0.005 for UDP-GlcNAc and *p* < 0.0005 for UDP-Glc). These mutations, in the region of the C-terminal domain remodeled on ligand binding, were the only ones to introduce sugar-donor binding promiscuity to *Lr*GtfC_100-23_ (Table 2 ▸). Introduction of promiscuity through glycoengineering has been reported for other GTs (Tytgat *et al.*, 2019 ▸; Williams *et al.*, 2007 ▸; Biswas & Thattai, 2020 ▸). As expected, wild-type *Lr*GtfC_53608_ protein showed no significant increase in melting temperature in the presence of UDP-GlcNAc relative to that seen when no ligand was present (*p* = 0.48) (Table 2 ▸, Supplementary Table S2). In contrast, *Lr*GtfC_53608_ P243S showed a significant positive thermal shift upon the addition of UDP-Glc (*p* < 0.005) but not with its cognate sugar donor UDP-GlcNAc. This was the only variant among those tested to show a reversed sugar-donor binding specificity profile. These results provide evidence that site-directed mutagenesis of single residues in the active site of *Lr*GtfCs could induce changes in UDP-sugar specificity.

These findings could not have been predicted from sequence alignments alone and it is likely that substrate specificity is dictated by the local environment surrounding these residues in the *Lr*GtfC crystal structure. For example, Leu174 in *Lr*GtfC_100-23_ is present as a leucine or a phenyl­alanine in all GtfCs from genome-sequenced *L. reuteri*strains, irrespective of their sugar specificity. Furthermore, this residue is a phenylalanine in *Sp*Gtf3_FW213_ and *Sa*GtfC (Supplementary Figure S12). In the *Lr*GtfC_100-23_ crystal structure, the side chain of residue Phe184 (Phe181 in *Lr*GtfC_53608_) is in van der Waals contact with Leu174 (Fig. 1 ▸*c*). This former residue, in turn, helps form a shallow pocket on the active-site surface in the region of a bound sugar moiety. Ser238 is not conserved across all *L. reuteri* GtfC homologs reported or predicted to possess UDP-Glc specificity. In the *Lr*GtfC_100-23_ crystal structure, Ser238 is found on the enzyme surface and is the first residue of the α-helix formed by residues 238–246 in the C-domain on ligand binding. Its C^α^ atom is 11 Å distant from the nearest atom of the donor sugar in its complex with the enzyme (the C atom of the sugar *N*-acetyl methyl group), yet the S238P mutation in *Lr*GtfC_100-23_ led to a gain of UDP-GlcNAc binding affinity, while the corresponding mutation P243S in *Lr*GtfC_53608_ led to a loss of UDP-GlcNAc binding affinity and a gain of affinity for the noncognate sugar. We note that sugar-binding promiscuity was generated through a W240C mutation in *Lr*GtfC_100-23_ but not through the reciprocal mutation in *Lr*GtfC_53608_. These responses are difficult to decipher based on the structural data presented here and support the idea of the complex role played by neighboring residues in this region in conferring substrate specificity.

## 4. Conclusion

The strain-specific glycosylation of SRRPs in *L. reuteri* strains 100-23C (from rat) and ATCC 53608 (from pig) is mediated by GtfCs. *Lr*GtfC_100-23_ shows specificity for UDP-Glc, while *Lr*GtfC_53608_ prefers UDP-GlcNAc as a sugar donor, yet the enzymes differ at only ten amino-acid positions. Here, we have used X-ray crystallography to determine the structural basis of *Lr*GtfC sugar-donor specificity. Detailed analysis of the refined crystal structures of *Lr*GtfC_100-23_ in the apo, UDP-bound and UDP-GlcNAc forms revealed conformational changes relating the noncognate substrate-bound and product-bound forms of the enzyme, and close structural homology with streptococcal aSec Gtfs. We presume that these conformational changes will also be present in the complex of the enzyme with the cognate sugar donor prior to hydrolysis. Using site-directed mutagenesis, we revealed the importance of residues in the C-terminal sugar-donor binding domain in determining sugar-donor binding specificity. Significantly, the P243S variant of *Lr*GtfC_53608_ showed reverse specificity for UDP-sugar, binding exclusively to UDP-Glc. Taken together, these results yielded novel insights into the structural basis for the strain-dependent UDP-sugar specificity of GtfC from the *L. reuteri* accessory secretion system, which can be harnessed for glycoengineering applications such as the production of glycoconjugates with bespoke glycosylation profiles.

## 5. Related literature

The following references are cited in the supporting information for this article: Edgar (2004 ▸), Kabsch & Sander (1983 ▸), Larkin *et al.* (2007 ▸) and Poirot *et al.* (2004 ▸).

## Supplementary Material

PDB reference: glycosyltransferase C, apo, 9htx

PDB reference: complex with UDP-GlcNAc, 9hu9

PDB reference: complex with UDP, 9hua

Supplementary Tables and Figures. DOI: 10.1107/S2059798325008782/jc5064sup1.pdf

## Figures and Tables

**Table 1:** *Lr*GtfC_100-23_ X-ray diffraction data-collection statistics Values in parentheses are for the highest resolution bin.

Ligand	Apo	UDP	UDP-GlcNAc
PDB entry	9htx	9hua	9hu9
Data collection			
Resolution (Å)	72.66–2.00 (2.07–2.00)	70.14–2.60 (2.69–2.60)	60.7–2.40 (2.49–2.40)
Space group	*P*2_1_2_1_2_1_	*P*12_1_1	*P*2_1_2_1_2_1_
Temperature (K)	100	100	100
*a*, *b*, *c* (Å)	70.44, 140.49, 145.32	72.41, 70.69, 140.31	76.64, 121.39, 174.10
α, β, γ (°)	90, 90, 90	90, 91.37, 90	90, 90, 90
Total reflections	2620719 (247437)	308060 (31127)	425334 (42545)
Unique reflections	98064 (9711)	43912 (4322)	64276 (6327)
Multiplicity	26.7 (25.5)	7.0 (7.2)	6.6 (6.7)
Completeness (%)	99.63 (99.19)	99.54 (99.19)	99.69 (97.99)
〈*I*/σ(*I*)〉	20.47 (1.26)	5.05 (1.18)	5.62 (0.58)
Wilson *B* factor (Å^2^)	40.63	49.39	46.80
*R*_merge_	0.112 (1.286)	0.202 (1.292)	0.236 (2.177)
*R*_meas_	0.114 (1.315)	0.219 (1.394)	0.256 (2.359)
*R*_p.i.m._	0.0229 (0.2734)	0.0826 (0.5217)	0.0990 (0.9018)
CC_1/2_	0.997 (0.781)	0.985 (0.909)	0.990 (0.516)
CC*	0.999 (0.936)	0.996 (0.976)	0.997 (0.825)
Structure refinement			
Reflections used in refinement	97703 (9632)	43715 (4287)	64083 (6200)
Reflections used for *R*_free_	5088 (499)	2152 (298)	3203 (288)
*R*_work_	0.2125 (0.4585)	0.2258 (0.3943)	0.1898 (0.3456)
*R*_free_	0.2556 (0.4656)	0.2788 (0.4107)	0.2234 (0.3382)
CC(work)	0.960 (0.594)	0.949 (0.780)	0.967 (0.720)
CC(free)	0.950 (0.544)	0.931 (0.680)	0.958 (0.556)
Non-H atoms			
Total	11001	10710	11218
Macromolecules	10810	10662	10879
Ligands	—	75 [UDP]	78 [UD1], 25 [UDP]
Solvent	165	48	192
Protein residues	1344	1316	1340
R.m.s.d., bond lengths (Å)	0.015	0.011	0.016
R.m.s.d., angles (°)	2.31	1.92	2.52
Ramachandran favored (%)	97.30	96.60	97.75
Ramachandran allowed (%)	2.40	2.55	1.80
Ramachandran outliers (%)	0.30	0.85	0.45
Rotamer outliers (%)	3.88	4.69	4.44
Clashscore	3.38	9.01	5.54
Average *B* factor (Å^2^)			
Overall	71.48	71.83	49.27
Macromolecules	71.79	71.94	49.05
Ligands	—	70.6 [UDP]	53.8 [UD1], 69.1 [UDP]
Solvent	50.72	50.85	42.25
No. of TLS groups	8	8	8

**Table 2:** Significance of melting-temperature shifts of *Lr*GtfC variants in the presence of UDP-sugars Recombinant wild-type (WT) and variant *Lr*GtfC_53608_ and *Lr*GtfC_100-23_ proteins at 20 µ*M* concentration in 50 m*M* Tris pH 7.5 analyzed by thermal shift assay in the absence or presence of 3 m*M* UDP-Glc or 3 m*M* UDP-GlcNAc (*n* = 4). Student’s *t*-test probability shown relative to *T*_m_ for the corresponding variant protein with no ligand added. Color code: *p* < 0.0005, green text; *p* < 0.005, amber text; *p* > 0.05, black text.

Strain	Variant	Ligand	
		UDP-Glc	UDP-GlcNAc
*Lr*GtfC_100-23_	WT	<0.0001	0.8733
	S238P	0.0002	0.0012
	W240C	0.0002	<0.0001
	L174F	<0.0001	0.7312
	D101A	0.0001	0.3632
*Lr*GtfC_53608_	WT	0.4792	<0.0001
	P243S	0.0041	0.1545
	C245W	0.1398	0.0008
	F179L	0.9758	0.0046
	D106A	0.1960	<0.0001

## Data Availability

Crystallographic data for the structures of *Lr*GtfC_100–23_ reported in this article have been deposited in the RCSB Protein Data Bank and are available free of charge via the DOIs https://doi.org/10.2210/pdb9HTX/pdb, https://doi.org/10.2210/pdb9HUA/pdb and https://doi.org/10.2210/pdb9HU9/pdb for the apo form, the complex with UDP and the complex with UDP-GlcNAc, respectively.
