# Supplementary material for: A structural basis for the strain-dependent UDP-sugar specificity of glycosyltransferase C from the Limosilactobacillus reuteri accessory secretion system
Source: Acta Crystallogr D Struct Biol. 2025 Nov 5;81(Pt 12):708–17. doi: 10.1107/S2059798325008782 (PMC12809243; doi:10.1107/S2059798325008782)
Supplement: Supplementary file 1 [file d-81-00708-sup1.pdf]

STRUCTURAL
BIOLOGY

**Volume 81 (2025)**

**Supporting information for article:**

# A structural basis for the strain-dependent UDP-sugar specificity of glycosyltransferase C from the *Limosilactobacillus reuteri* accessory secretion system

**Ryan Griffiths, Hans Pfalzgraf, Dimitris Latousakis, Gareth Ashworth, Changjiang Dong, Andrew Hemmings and Nathalie Juge**

**Table S1** Primers and targets for site-directed mutagenesis of *Lr*GtfCs.

| Primer (5'-3') | Template | Annealing Temperature/ °C | Site-Directed Mutagenesis Target |
|---|---|---|---|
| F: ggtaattaatcttgctggtaacc | *Lr*GtfC$_{53608}$-pOPINF | 60 | *Lr*GtfC$_{53608}$ F179L |
| R: ccaatggtcgttctaattaatgg | | | |
| F: gtcaaatgagagttattgtaaaa | | | *Lr*GtfC$_{53608}$ P243S |
| R: ttttacaataactctcatttgac | | | |
| F: tgagccatattggaaaaactacatg | | | *Lr*GtfC$_{53608}$ C245W |
| R: catgtagtttttccaatatggctca | | | |
| F: ggtaattaattttgctggtaacc | *Lr*GtfC$_{100-23}$-pET28a | | *Lr*GtfC$_{100-23}$ L179F |
| R: ggttaccagcaaaattaattacc | | | |
| F: tggtcaaatgagccatattggaaaaac | | | *Lr*GtfC$_{100-23}$ S238P |
| R: gtttttccaatatggctcatttgacca | | | |
| F: tggtttagtatgttcaaatgagtc | | | *Lr*GtfC$_{100-23}$ W240C |
| R: gactcatttgaacatactaaacca | | | |

**Table S2** Melting temperatures of *Lr*GtfC variants in the presence of UDP sugars[a].

| | | $T_m$°C | | |
|---|---|---|---|---|
| | **Variant** | **No ligand** | **UDP-Glc** | **UDP-GlcNAc** |
| *Lr**GtfC**$_{100-23}$* | WT | 48.32 ± 0.2 | 53.34 ± 0.2 | 48.35 ± 0.3 |
| | S238P | 47.38 ± 0.1 | 50.11 ± 0.7 | 48.29 ± 0.3 |
| | W240C | 48.51 ± 0.4 | 51.14 ± 0.5 | 51.23 ± 0.2 |
| | L174F | 48.42 ± 0.3 | 51.32 ± 0.4 | 48.33 ± 0.1 |
| | D101A | 48.13 ± 0.2 | 51.33 ± 0.4 | 48.35 ± 0.4 |
| *Lr**GtfC**$_{53608}$* | WT | 48.27 ± 0.3 | 48.70 ± 1.1 | 51.32 ± 0.4 |
| | P243S | 48.11 ± 1.2 | 51.41 ± 0.8 | 49.14 ± 0.4 |
| | C245W | 48.15 ± 0.3 | 49.12 ± 1.1 | 52.30 ± 1.3 |
| | F179L | 48.26 ± 0.4 | 48.24 ± 1.2 | 50.63 ± 1.0 |
| | D106A | 48.27 ± 0.3 | 48.5 ± 0.1 | 50.99 ± 0.4 |

[a]Recombinant wild type (WT) and variant *Lr*GtfC$_{53608}$ and *Lr*GtfC$_{100-23}$ proteins in 50 mM Tris pH 7.5 analysed by thermal shift assay in the absence or presence of 3 mM UDP-Glc or 3 mM UDP-GlcNAc. n=4, errors shown as standard error of the mean (SEM), rounded to 1 DP.

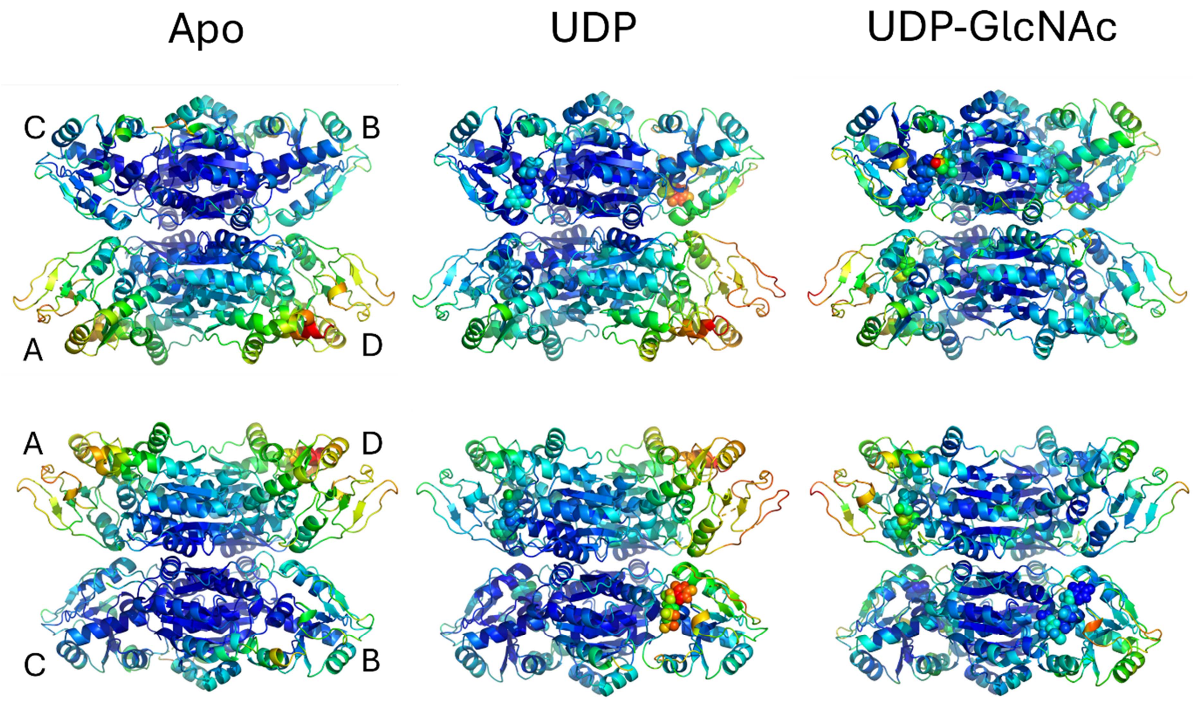


**Figure S1** A representation of temperature factors in refined *Lr*GtfC$_{100\text{-}23}$ structures. Upper row: Front views of the tetramers least squares-aligned according to the Cα atom coordinates of the A-chain and coloured by B-factor at the Cα atom. Polypeptide chains are labelled. Atoms of bound UDP and UDP-GlcNAc ligands are shown as spheres coloured by B-factor. Lower row: Rear views of the structures. obtained by rotating the structures in the upper low by 180° about the horizontal.

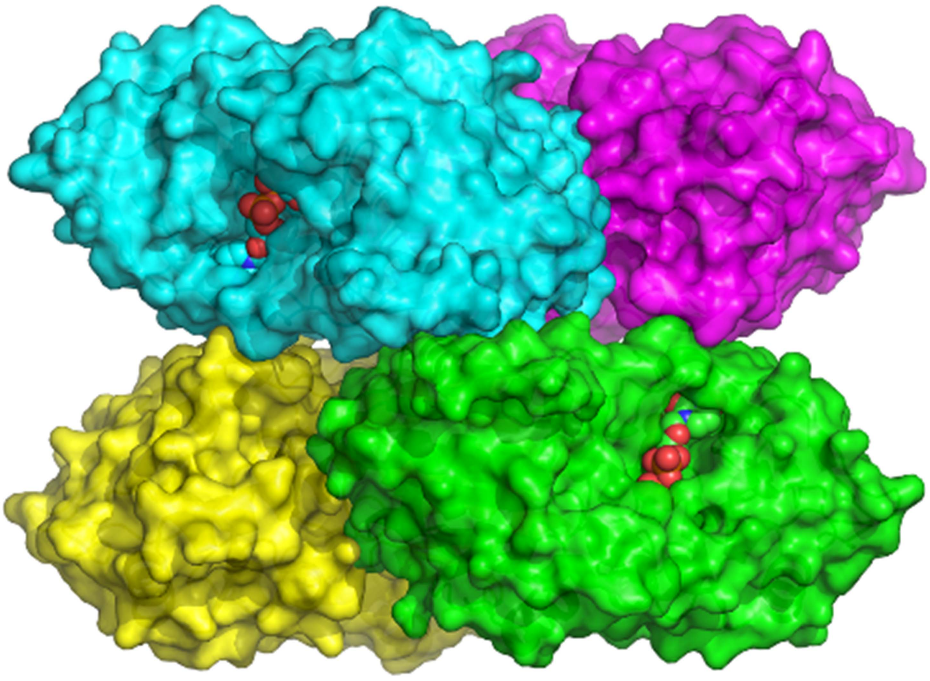

**Figure S2** The *Lr*GtfC$_{100-23}$ tetramer. An *Lr*GtfC$_{100-23}$ tetramer formed from two dimers (chains A, D and B, C, respectively) as found in the asymmetric unit of crystals of its complex with UDP-GlcNAc. A molecular surface rendering is shown with chains coloured green (chain A), cyan (chain B), magenta (chain C) and yellow (chain D). In monomers A and B, a bound UDP-GlcNAc molecule is partially visible with atoms shown as spheres and oxygen coloured red, nitrogen green and carbon green.

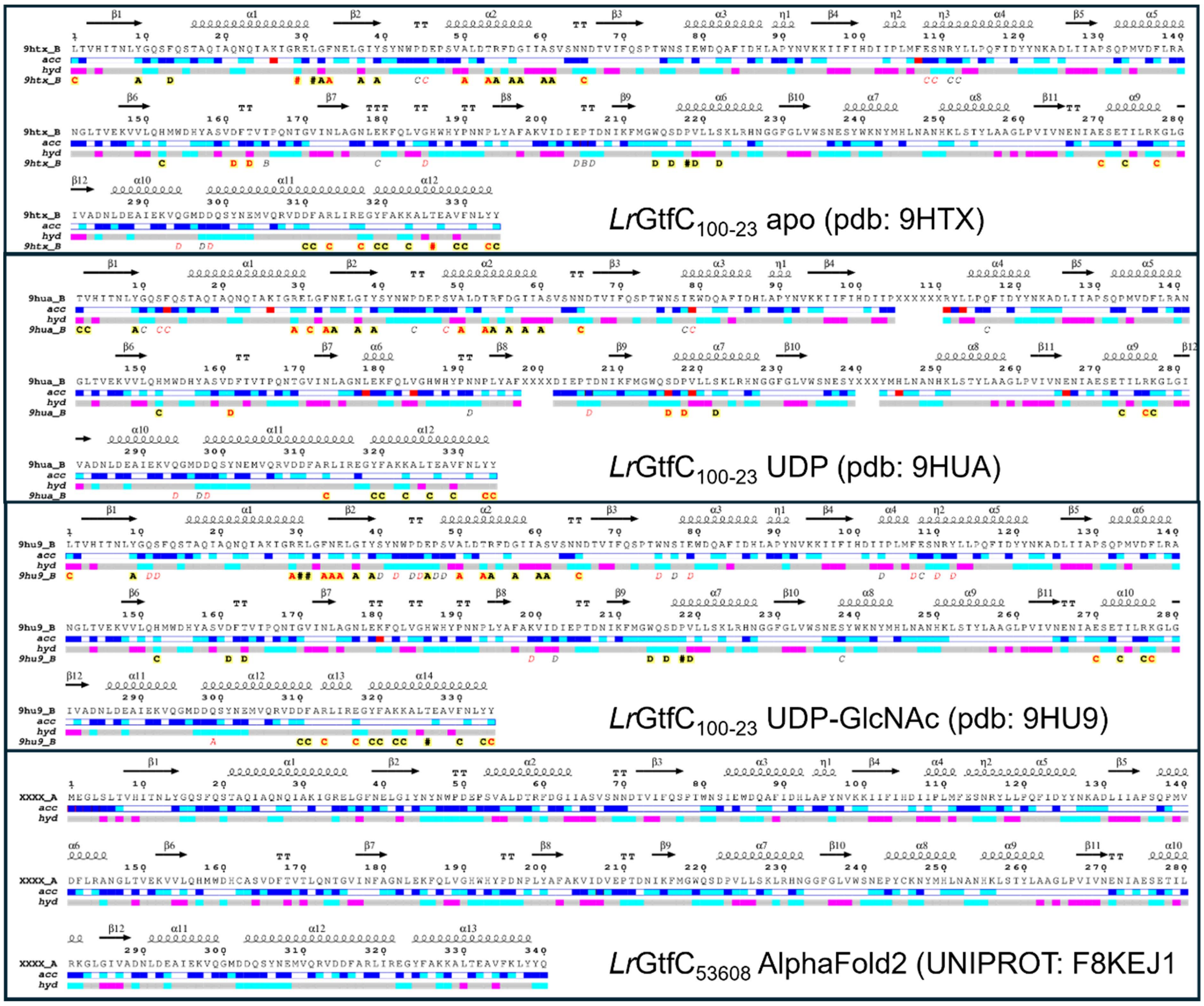


**Figure S3** Secondary structures observed in crystal structures of *Lr*GtfC$_{100\text{-}23}$ and in the AlphaFold2-predicted structure of *Lr*GtfC$_{53608}$. The secondary structures shown are calculated from the coordinates of chain B in the *Lr*GtfC$_{100\text{-}23}$ structures. The panels carry annotation indicating their respective PDB or AlphaFold Protein Structure Database entry codes.

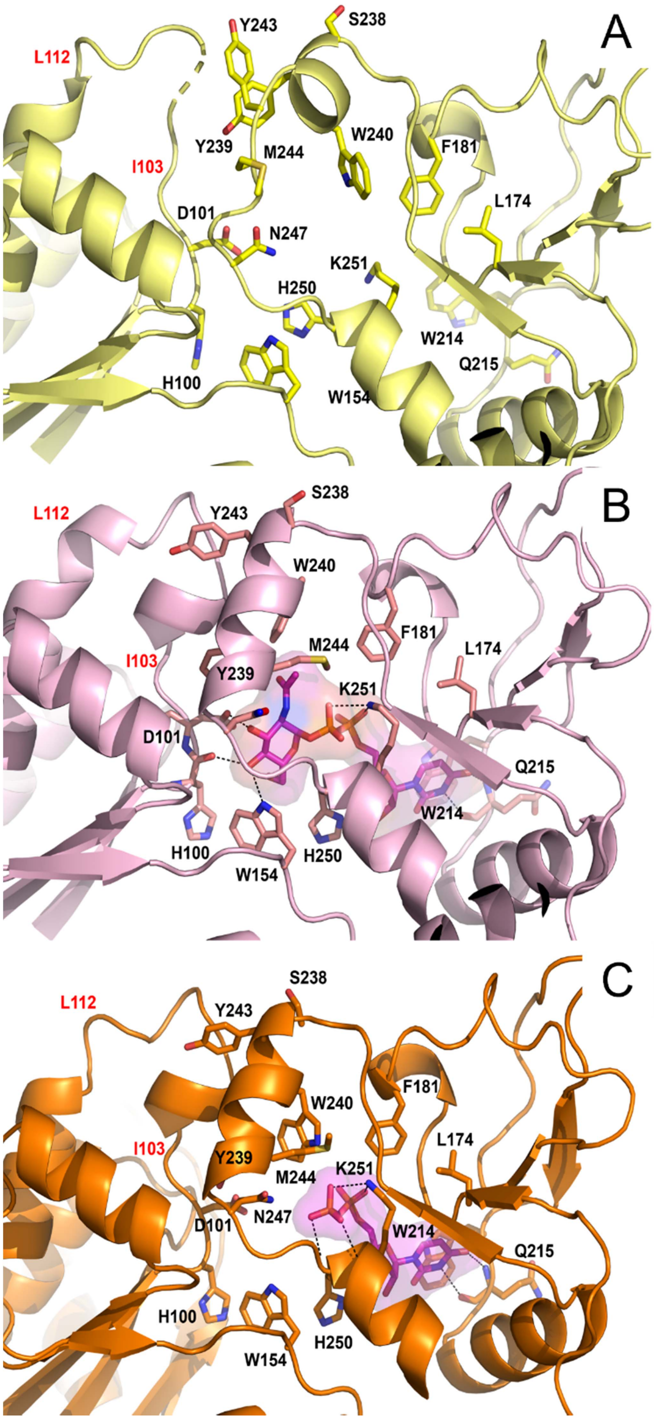


**Figure S4** Alternate views of apo- and ligand-bound forms of *Lr*GtfC$_{100\text{-}23}$. (**A**) The apo enzyme (PDB:9HTX). Residues 103 and 112 (red text) delimit a flexible region of polypeptide adjacent to the binding site in the apo-structure. (B) The UDP-GlcNAc-bound enzyme (PDB: 9HU9). (C) The UDP-bound enzyme (PDB: 9HUA). In panels (B,C) bound UDP and UDP-GlcNAc are shown in stick format with molecular surface shown. For each structure selected residues are shown in stick format and with a selection of hydrogen bond interactions indicated by black dashed lines.

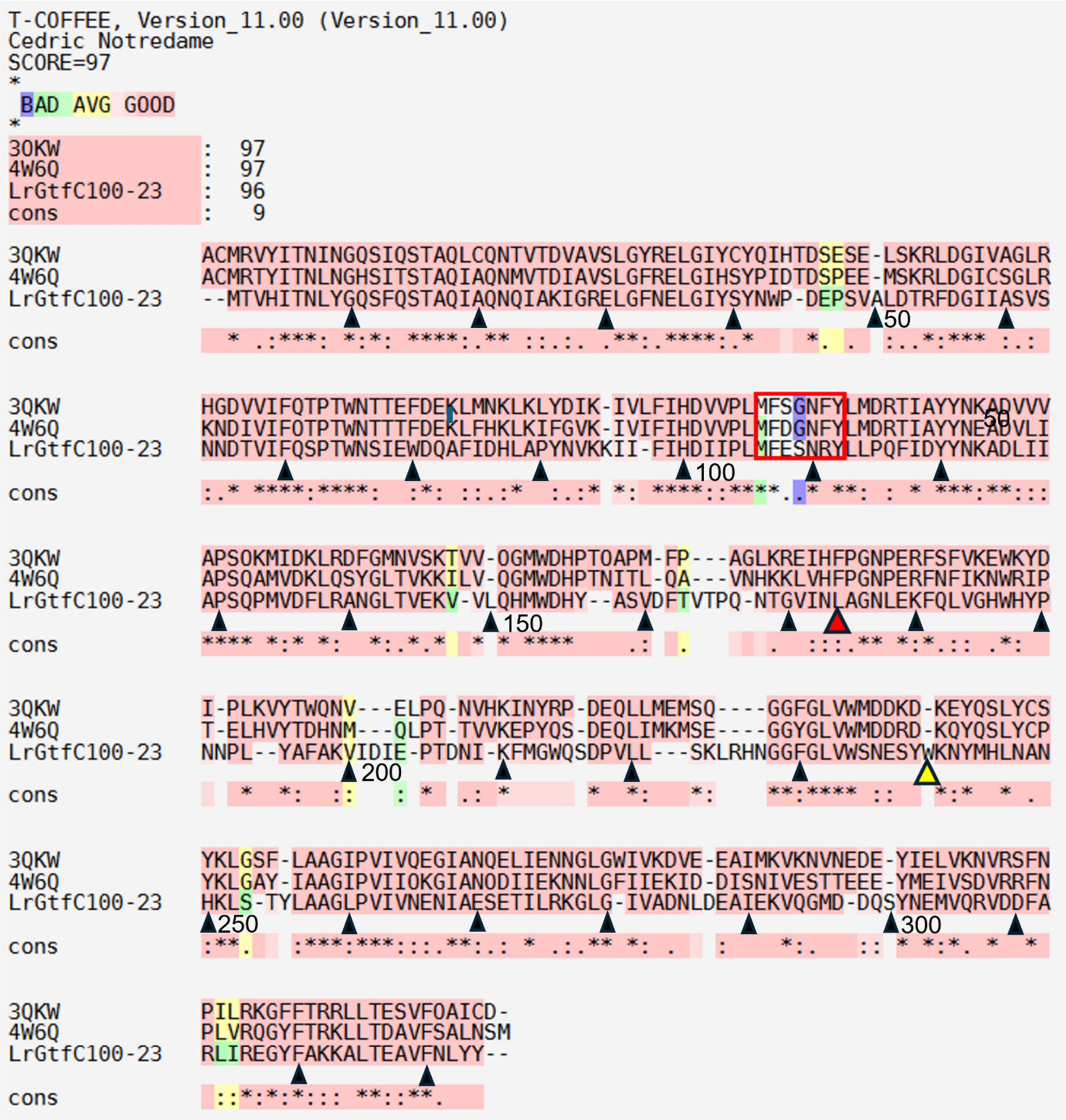

**Figure S5** Structure-based sequence alignment of *Lr*GtfC$_{100\text{-}23}$ with two closely related glucosyl transferases**.** Sequences are taken from the GT113 family enzymes Gtf3 from *S. parasanguinis* FW213 (PDB: 3QKW) (SpGtf3$_{FW213}$) and GtfC from *S. agalactiae* (PDB: 4W6Q) (SaGtfC). Alignment performed using 3D-Coffee version 11.00 (Poirot et al, 2004). The *Lr*GtfC$_{100\text{-}23}$ MFESNR sequence corresponds to *Sa*GtfC MFDGNF and these residues are indicated by a red box. Residues L174 and W240 of LrGtfC$_{100\text{-}23}$ are indicated by red and yellow triangles, respectively. Every thenth residue of *Lr*GtfC$_{100\text{-}23}$ is marked by a black triangle and with every 50th residue numbered.

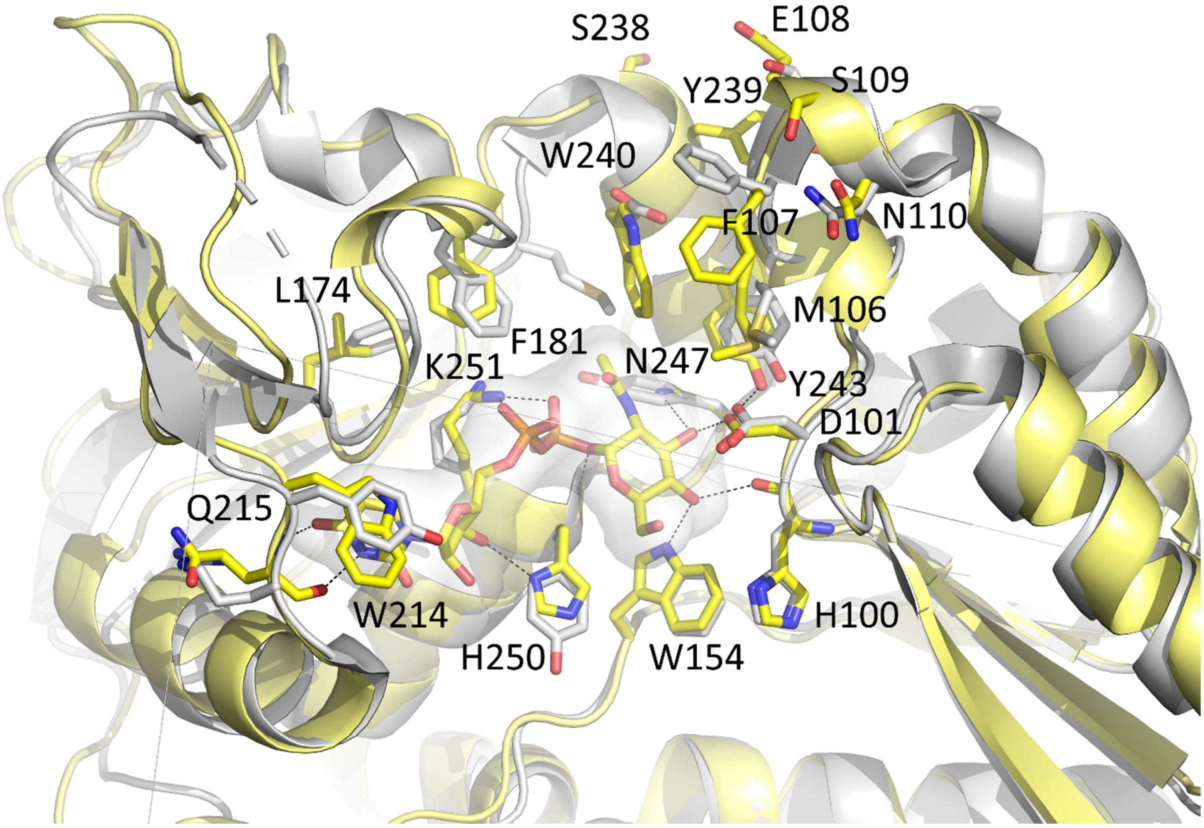


**Figure S6** Comparison of the crystal structures of *Lr*GtfC$_{100\text{-}23}$ and *Sp*Gtf3$_{FW213}$. A superposition of the UDP-GlcNAc bound *Lr*GtfC$_{100\text{-}23}$ (yellow cartoon, yellow carbon atoms) and UDP-bound (grey cartoon, grey carbon atoms) *Sp*Gtf3$_{FW213}$ structures. UDP-GlcNAc is shown in stick format with a molecular surface rendering. Selected active site residues are shown in stick format and hydrogen bond interactions indicated by black dashed lines. Residues from the *Lr*GtfC$_{100\text{-}23}$ structure are labelled where shown.

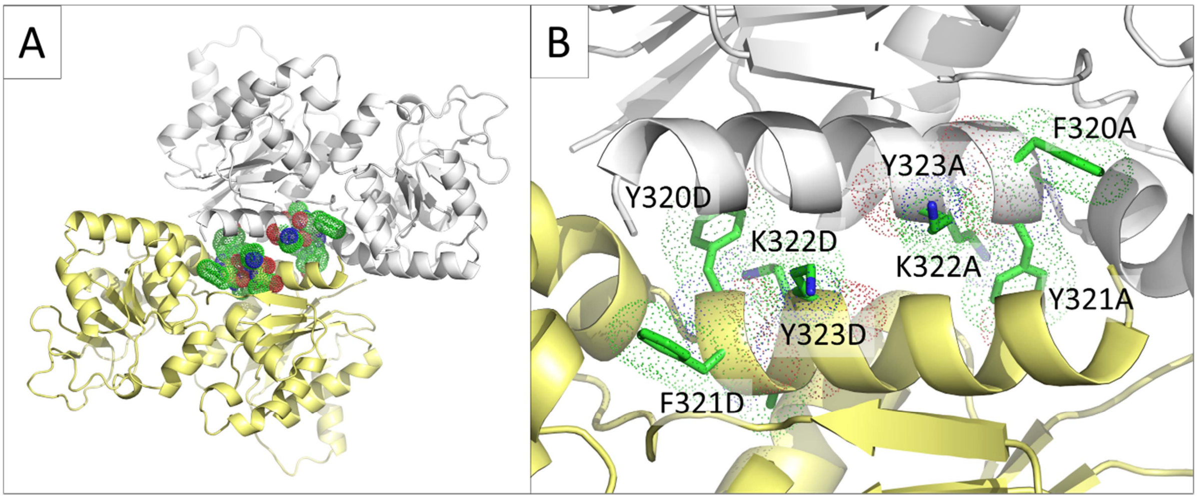


**Figure S7** Residues at the *Lr*GtfC$_{100\text{-}23}$ dimer interface. Residues Phe320, Tyr321, Lys322 and Lys323 at the interface of the A and D chains of the crystal structure of *Lr*GtfC$_{100\text{-}23}$. Cartoon representations of the A (grey) and D (yellow) chains are shown. Residues are shown in stick format and coloured according to atom type (carbon-green) and labelled. Dot surfaces are shown for the chosen interface residues.

```
LrGtfC100-23      LTVHITNLYGQSFQSTAQIAQNQIAKIGRELGFNELGIYSYNWPDEPSVALDTRFDGIIA
LrGtfC53608       MTVHITNLYGQSFQSTAQIAQNQIAKIGRELGFNELGIYNYNWPDEPSVALDTRFDGIIA
                  :**************************************.********************

LrGtfC100-23      SVSNNDTVIFQSPTWNSIEWDQAFIDHLAPYNVKKIIFIHDIIPLMFESNRYLLPQFIDY
LrGtfC53608       SVSNNDTVIFQSPTWNSIEWDQAFIDHLAPYNVKKIIFIHDIIPLMFESNRYLLPQFIDY
                  ************************************************************

LrGtfC100-23      YNKADLIIAPSQPMVDFLRANGLTVEKVVLQHMWDHYASVDFTVTPQNTGVINLAGNLEK
LrGtfC53608       YNKADLIIAPSQPMVDFLRANGLTVEKVVLQHMWDHCASVDFTVTLQNTGVINFAGNLEK
                  ************************************ ******** *******:******

LrGtfC100-23      FQLVGHWHYPNNPLYAFAKVIDIEPTDNIKFMGWQSDPVLLSKLRHNGGFGLVWSNESYW
LrGtfC53608       FQLVGHWHYPDNPLYAFAKVIDVEPTDNIKFMGWQSDPVLLSKLRHNGGFGLVWSNEPYC
                  **********:***********:**********************************.* 

LrGtfC100-23      KNYMHLNANHKLSTYLAAGLPVIVNENIAESETILRKGLGIVADNLDEAIEKVQGMDDQS
LrGtfC53608       KNYMHLNANHKLSTYLAAGLPVIVNENIAESETILRKGLGIVADNLDEAIEKVQGMDDQS
                  ************************************************************

LrGtfC100-23      YNEMVQRVDDFARLIREGYFAKKALTEAVFNLYY-
LrGtfC53608       YNEMVQRVDDFARLIREGYFAKKALTEAVFKLYYQ
                  ******************************:*** 
```

**Figure S8** Alignment of *Lr*GtfC$_{100\text{-}23}$ and *Lr*GtfC$_{53608}$ amino acid sequences. Alignment produced by Clustal W (Larkin et al, 2007).

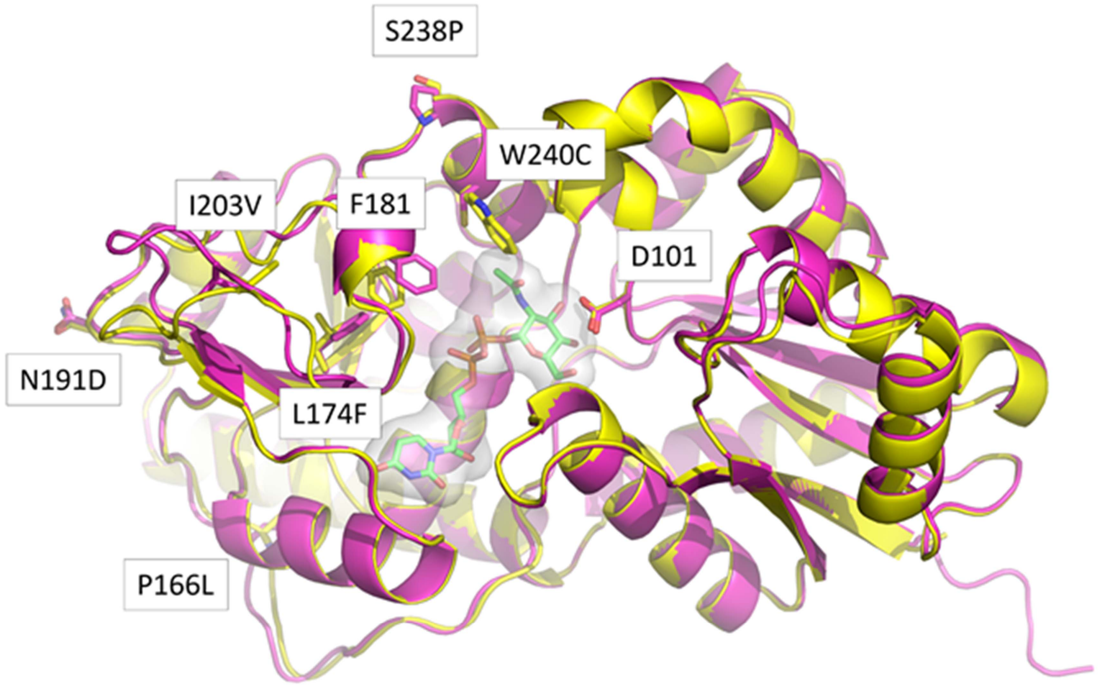


**Figure S9** A comparison of the crystal structure of *Lr*GtfC$_{100\text{-}23}$ and the Alphafold2-predicted structure of $_{\mathrm{Lr}}$GtfC$_{53608}$. Structures are shown in cartoon format with *Lr*GtfC$_{100\text{-}23}$ in yellow and *Lr*GtfC$_{53608}$ in magenta. The position of the bound ligand in the crystal structure is shown in stick format with a molecular surface rendering in grey. The sidechains of residues in the UDP-sugar binding domain of *Lr*GtfC$_{100\text{-}23}$ which differ from those in *Lr*GtfC$_{53608}$ are shown in stick representation and the corresponding substitution labelled. The sidechains of residues D101, the presumed catalytic aspartate, and F184, in van der Waals contact with L174, in *Lr*GtfC$_{100\text{-}23}$ (D106 and F181, respectively, in *Lr*GtfC$_{53608}$) are also shown and labelled.

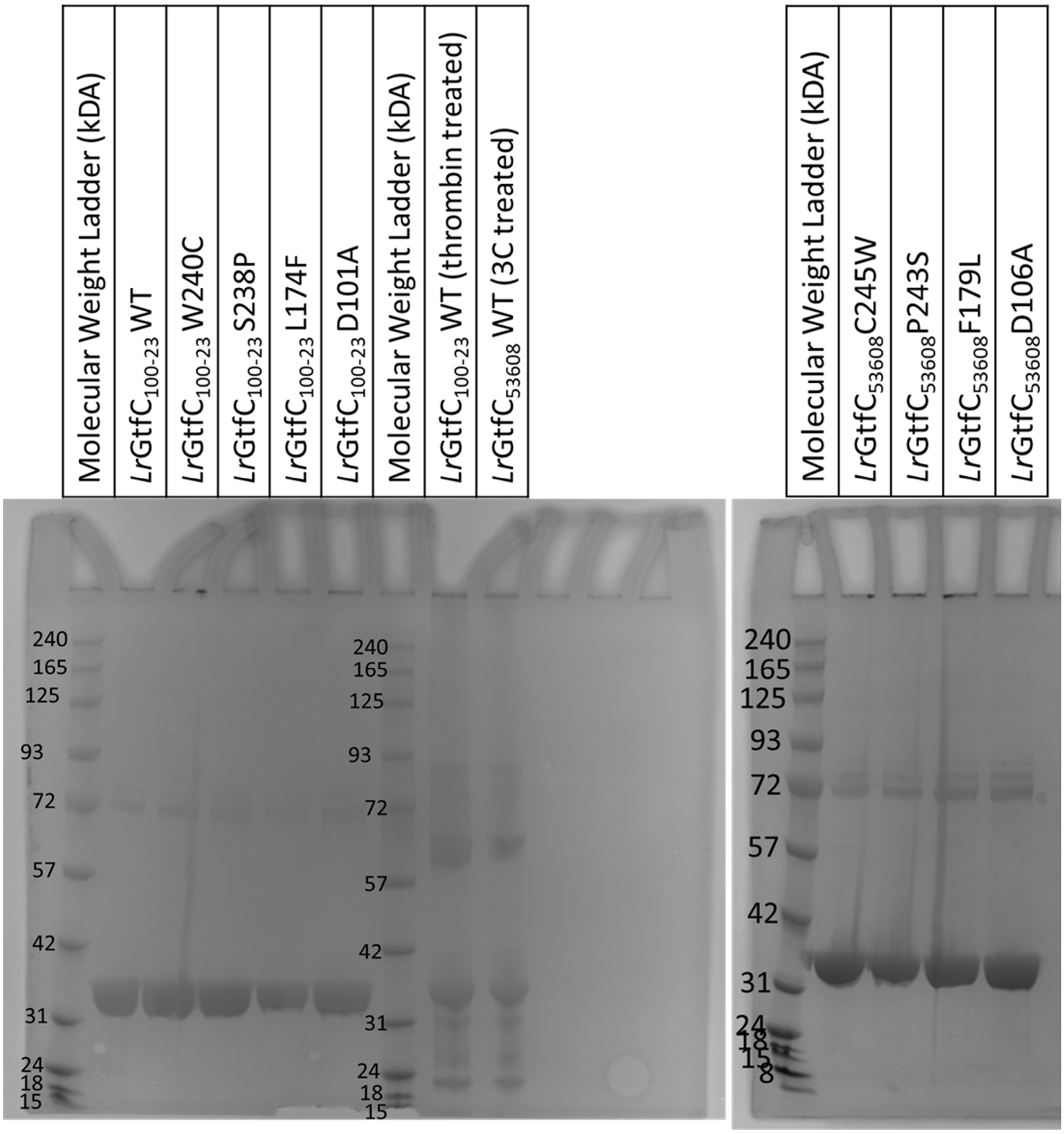


**Figure S10**SDS-PAGE analysis of purified recombinant GtfC variants from *L. reuteri* strains ATCC 53608 and 100-23. Recombinant *Lr*GtfC$_{100\text{-}23}$ and *Lr*GtfC$_{53608}$ wild type and mutant proteins were expressed intracellularly in *E. coli* BL21(DE3) and purified by NiNTA affinity chromatography. His-tag removal was performed for the WT proteins using thrombin or 3C-protease for *Lr*GtfC$_{100\text{-}23}$ and *Lr*GtfC$_{53608}$, respectively. The expected molecular weight is around 39 kDa for *Lr*GtfC derived from both strains.

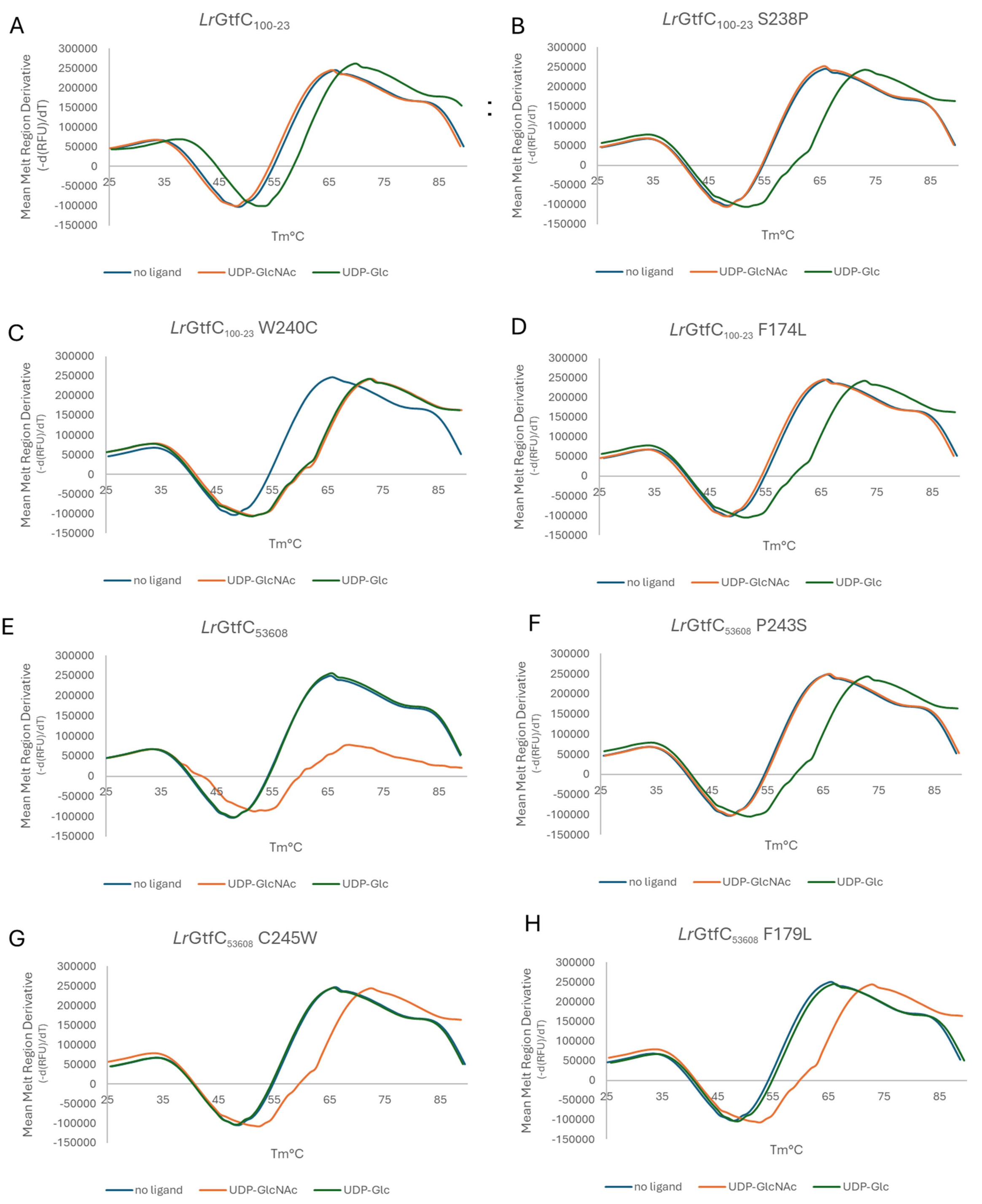


**Figure S11** Thermal shift analysis of *Lr*GtfC variants. (A) *Lr*GtfC$_{100\text{-}23}$WT (B) *Lr*GtfC$_{100\text{-}23}$S238P (C) *Lr*GtfC$_{100\text{-}23}$W240C (D) *Lr*GtfC$_{100\text{-}23}$L174F (E) *Lr*GtfC$_{53608}$WT (F) *Lr*GtfC$_{53608}$P243S (G) *Lr*GtfC$_{53608}$C245W (H) *Lr*GtfC$_{53608}$F174L. Recombinant wild type and variant *Lr*GtfC$_{53608}$ and *Lr*GtfC$_{100\text{-}23}$ proteins in 50 mM Tris pH 7.5 were analysed by thermal shift analysis in the absence or presence of 3mM UDP-Glc or 3 mM UDP-GlcNAc.

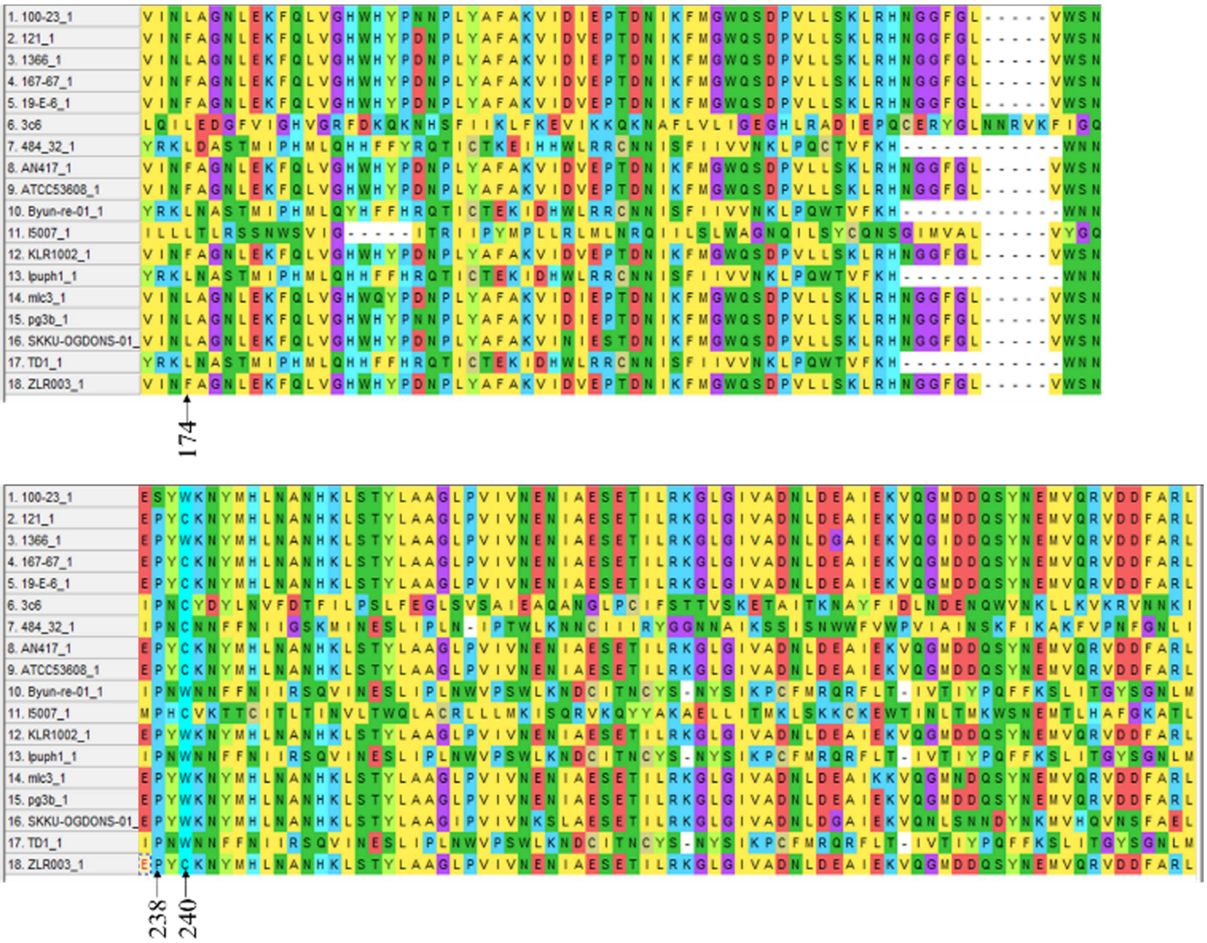


**Figure S12** MUSCLE alignment of *Lr*GtfC amino acid sequences. *Lr*GtfC amino acid sequences (shown on left) from 18 *L. reuteri* strains were aligned using MUSCLE (Edgar, 2004). Residues between positions 172 and 315 are shown with arrows indicating amino acids at position 174, 238 and 240.

**Supplementary References**

Edgar, RC (2004) MUSCLE: multiple sequence alignment with high accuracy and high throughput. Nucleic Acids Res. 32(5), 1792–1797.

Holm, L. (2022) Dali server: structural unification of protein families. Nucleic Acids Res. 50 (W1), W210–W215.

Kabsch W, Sander C (1983) Dictionary of protein secondary structure: pattern recognition of hydrogen-bonded and geometrical features. Biopolymers. 22, 2577-2637.

Larkin MA, Blackshields G, Brown NP, Chenna R, McGettigan PA, McWilliam H, Valentin F, Wallace IM, Wilm A, Lopez R, Thompson JD, Gibson TJ, Higgins DG (2007). Clustal W and Clustal X version 2.0. Bioinformatics. 23, 2947-2948.

Poirot O, Suhre K, Abergel C, O'Toole E, Notredame C. 3DCoffee@igs: a web server for combining sequences and structures into a multiple sequence alignment. Nucleic Acids Res. 2004 Jul 1;32(Web Server issue): W37-40.
